# Cytokine and Chemokine Expression in Kidneys during Chronic Leptospirosis in Reservoir and Susceptible Animal Models

**DOI:** 10.1371/journal.pone.0156084

**Published:** 2016-05-24

**Authors:** Mariko Matsui, Louise Roche, Sophie Geroult, Marie-Estelle Soupé-Gilbert, Didier Monchy, Michel Huerre, Cyrille Goarant

**Affiliations:** 1 Institut Pasteur International Network, Institut Pasteur de Nouvelle-Calédonie, Leptospirosis Research and Expertise Unit, Noumea, New Caledonia; 2 Anatomic Pathology Laboratory, Gaston-Bourret Territorial Hospital Center, Noumea, New Caledonia; 3 Unité de Recherche et Expertise en Histotechnologie et Pathologie, Institut Pasteur, Paris, France; 4 Departement de Pathologie, Institut Curie, Paris, France; University of Toledo College of Medicine and Life Sciences, UNITED STATES

## Abstract

Leptospirosis is caused by pathogenic spirochetes of the genus *Leptospira*. Humans can be infected after exposure to contaminated urine of reservoir animals, usually rodents, regarded as typical asymptomatic carriers of leptospires. In contrast, accidental hosts may present an acute form of leptospirosis with a range of clinical symptoms including the development of Acute Kidney Injury (AKI). Chronic Kidney Disease (CKD) is considered as a possible AKI-residual sequela but little is known about the renal pathophysiology consequent to leptospirosis infection. Herein, we studied the renal morphological alterations in relation with the regulation of inflammatory cytokines and chemokines, comparing two experimental models of chronic leptospirosis, the golden Syrian hamster that survived the infection, becoming carrier of virulent leptospires, and the OF1 mouse, a usual reservoir of the bacteria. Animals were monitored until 28 days after injection with a virulent *L*. *borgpetersenii* serogroup Ballum to assess chronic infection. Hamsters developed morphological alterations in the kidneys with tubulointerstitial nephritis and fibrosis. Grading of lesions revealed higher scores in hamsters compared to the slight alterations observed in the mouse kidneys, irrespective of the bacterial load. Interestingly, pro-fibrotic TGF-β was downregulated in mouse kidneys. Moreover, cytokines IL-1β and IL-10, and chemokines MIP-1α/CCL3 and IP-10/CXCL-10 were significantly upregulated in hamster kidneys compared to mice. These results suggest a possible maintenance of inflammatory processes in the hamster kidneys with the infiltration of inflammatory cells in response to bacterial carriage, resulting in alterations of renal tissues. In contrast, lower expression levels in mouse kidneys indicated a better regulation of the inflammatory response and possible resolution processes likely related to resistance mechanisms.

## Introduction

Leptospirosis is a neglected widespread zoonosis caused by pathogenic spirochetes of the genus *Leptospira*. More than one million human cases of leptospirosis are estimated to occur worldwide annually with the highest incidence in the African, Asia-Pacific, Latin America and Caribbean regions [1]. Pathogens are transmitted to humans by direct or indirect contact with contaminated urine from infected Mammals, and animals are most frequently divided into maintenance or reservoir, and incidental hosts as humans [2].

Severe cases of human leptospirosis present a range of symptoms including fever, multiple organ failures with renal and hepatic insufficiency, and pulmonary manifestations, possibly leading to death [2]. Kidney injury is an early manifestation of acute leptospirosis and Acute Kidney Injury [AKI; formerly named acute renal failure [3]] is commonly reported in leptospirosis [4] with a mean incidence of 36%, of which 12% die as a results [5]. Moreover, oliguria constitutes an important risk factor for fatality in leptospirosis [6]. Leptospirosis-related AKI is characterized by acute tubular necrosis and interstitial nephritis with tubular degeneration and interstitial oedema with cellular infiltration of mononuclear cells [7]. Recovery of renal functions from leptospirosis-related renal failures may take several months [8]. Interestingly, chronic kidney disease (CKD) was suggested to be a possible long-term outcome of the leptospirosis-related AKI regarding results of a cohort study conducted in Sri Lanka showing that 9% of patients developed CKD [9]. In this study, renal biopsies were done in two patients presenting persistent abnormal renal functions and revealed inflammatory infiltrates, tubular atrophy and interstitial fibrosis. Leptospirosis-induced end-stage renal failure (ESRF) was also reported with tubular atrophy and interstitial fibrosis observed in kidney biopsy [10]. Asymptomatic cases of human leptospirosis were also shown by serological or molecular analysis [11, 12], and prolonged urinary shedding of spirochetes was reported [13, 14] associated with asymptomatic renal colonization by both pathogenic or intermediate leptospires [15]. Moreover, recent epidemiological survey in Taiwan revealed that chronic human exposure to leptospirosis was associated with prevalence and severity of CKD and highlighted asymptomatic leptospirosis as an overlooked risk for CKD [16]. Interestingly, recent epidemic of CKD of unknown etiology named Mesoamerican Nephropathy has emerged in Central America [17] and was hypothesized to be related to infection with pathogens as Hantavirus and *Leptospira* [18]. Contribution of leptospirosis is also evoked as a possible origin for CKD development with unidentified origin in Asia Pacific region [19]. Thus, better understanding of physiopathological processes involved in the development of renal failures and possible CKD appearance related to leptospirosis sequelae is of importance.

Hamsters and guinea pigs are the standard models used to produce an acute infection modeling severe human leptospirosis [2]. However, these particular animals can also face chronic leptospirosis after experimental infection with *L*. *interrogans* Pomona or Grippotyphosa [20, 21] or *L*. *borgpetersenii* Hardjo or Ballum [22, 23]. In contrast, rats and mice are considered as major maintenance hosts, and different host–serovar associations seem to be ubiquitous as observed for rats (commonly *Rattus norvegicus* and *Rattus rattus*) with serogroup Icterohaemorrhagiae, and mice (*Mus musculus* and other *Mus* species) with the serogroup Ballum [24]. Others mammals are also considered as reservoirs of virulent leptospires, as cattle with the serovars Hardjo or Pomona, and dogs with the serovar Canicola. Carrier hosts mostly present asymptomatic leptospirosis, and the subsequent clearance of the pathogens from all organs except the kidneys is related to the urinary shedding of the bacteria [25]. Indeed, bacteria are maintained in the renal proximal tubules and excreted in the urine for several months. Renal lesions during chronic leptospirosis were reported in reservoir animals as dogs, rats, pigs and cattle infected with their associated leptospires [25]. Though asymptomatic, carrier hosts showed morphological changes of kidneys as observed in experimentally infected rats or wild infected carnivores mainly presenting chronic interstitial nephritis [26–28].

Pathogenesis of renal dysfunction and development of kidney injury during leptospirosis still needs to be clarified. Considering host-pathogen interaction aspect, leptospiral outer membrane proteins (OMPs) were shown to activate important transcription factor as the nuclear transcription factor kappa B (NF-κB) and the activator protein-1 (AP-1) in medullary thick ascending limb cells or in proximal tubules isolated from mice [29, 30]. Consequently, downstream genes, including the pro-inflammatory cytokine tumor necrosis factor-α (TNF-α) and the chemokine monocyte chemoattractant protein-1 (MCP-1/C-C-type chemokine ligand 2, CCL2), were overexpressed, and it was hypothesized that cellular damage in renal tissue could be related to the induction of these inflammatory mediators through the NF-κB signaling pathway. Interestingly, expression of the inducible nitric oxide synthase (iNOS) is also induced by inflammatory TNF-α [31], and nitric oxide produced by iNOS seems to have beneficial effects in ureteral obstruction [32] reported in leptospirosis-related AKI in human case [33]. Moreover, triggering of iNOS up-regulation and the resulting NO production seems crucial in the survival against acute leptospirosis as injection of iNOS inhibitor increased the mortality rate *in vivo* during experimental infection [34]. In contrast, *Leptospira*-induced renal fibrosis has been shown to be reduced in transgenic C57BL/6J mice lacking iNOS enzyme, suggesting that the regulation of nitric oxide pathway could participate in the induction of renal fibrosis [35]. In this latter study, expression of renal Transforming Growth Factor (TGF)-β was also investigated as being one of the most potent inducers of extracellular matrix and involved in development of renal fibrosis [36] but no regulation was shown.

Herein, we aim at studying regulation of major inflammatory cytokine gene expression in relation with development of renal lesions during chronic leptospirosis. Using the comparison between experimentally infected classical reservoir, the mouse, and unusual carrier animal, the golden Syrian hamster, we studied the regulation of the expression of pro-fibrotic TGF-β, of the inflammatory iNOS and cytokines interleukin-(IL-)1β, IL-10 and TNF-α, and of the chemokines gamma interferon-inducible protein 10 kDa (IP-10/ C-X-C-type chemokine ligand 10, CXCL10) and macrophage inflammatory protein-1 (MIP-1α/C-C-type chemokine ligand 3, CCL3) in the kidneys of animals that became chronic carriers of virulent leptospires. Concomitant development of renal inflammatory lesions, fibrosis and bacterial load were also studied. Interestingly, chronic carriage of *Leptospira* was characterized by significant differences in cytokine and chemokine gene expression profiles depending on animal models that could explain the differential and pronounced progression of renal lesions observed in hamsters compared to mice.

## Materials and Methods

### Animals and ethical concerns

OF1 mice (*M*. *musculus*) and golden Syrian hamsters (*Mesocricetus auratus*), whose genitors originated from Charles River Laboratories were bred in a restricted-access room at Institut Pasteur in New Caledonia. Animal manipulations were conducted according to the guidelines of the Animal Care and Use Committees of the Institut Pasteur of Paris and of New Caledonia, and followed European Recommendation 2007/526/EC. Protocols and experiments were approved by the Animal Care and Use Committees of the Institut Pasteur in New Caledonia.

### Strain of leptospires

Virulent *Leptospira borgpetersenii* serogroup Ballum isolate B3-13S was cultured from kidneys of a wild mouse (*M*. *musculus*) captured in 2009 in New Caledonia [37]. Characterization of its virulence was previously described, and intraperitoneal injection of 1 x 10^8^ bacteria led to chronic carriage of leptospires until 28 days postinfection [23]. Leptospires were cultured in liquid Ellinghausen-McCullough-Johnson-Harris (EMJH) medium at 30°C under aerobic conditions [38]. The bacterial cell concentration was determined using a Petroff-Hausser counting chamber (Hausser Scientific). Virulence was maintained by monthly passages in hamsters after intraperitoneal injection before re-isolation from blood.

### Experimental infections

Animals were individually housed in plastic cages with wood shaving bedding. Six- to eight-week-old hamsters or mice were injected intraperitoneally with B3-13S at 1 x 10^8^ leptospires in 500 to 800 μL EMJH medium for establishment of chronic infection. Non-infected animals were injected an equal volume of sterile EMJH and served as control animals. Survival and body weight were monitored during the experiments and animals were observed for any signs of clinical illness (loss of appetite or reactivity, prostration, ruffled fur, external hemorrhage). Animals were killed by atlanto-occipital dislocation after anesthesia with chloroform or sacrificed by inhalation of CO_2_ when moribund or at 14, 21 or 28 days postinfection. Blood from infected animals was collected in EDTA tube by cardiac puncture and rapidly processed for quantification of bacteremia. After dissection, tissues (kidneys, lungs, liver) were rapidly collected for molecular bioassays (within 5 minutes of euthanasia) and for histology. For extraction of total DNA from organs, ca. 25 mg samples were placed into MagNA Lyser Green Beads tubes (Roche Applied Sciences) containing 360 μL lysis buffer (QIAamp DNA Mini kit, Qiagen) and stored at 4°C until extraction. For total RNA, tissue samples were stored in 1,500 μL RNA*later* (Sigma-Aldrich) for stabilization of nucleic acids at room temperature for 2 h before conservation at -20°C until extraction. For histology, kidneys were fixed in 10% buffered formalin for 24 to 48 h and paraffin-embedded.

### Histology and staining

Three-μm serial sections were stained with hematoxylin-erythrosin (HE) and Masson’s trichrome stain to show collagen structures typical of fibrosis [39]. Morphological changes in renal tissue were described according to criteria commonly reported for acute or chronic leptospirosis in animal models, i.e. haemorrhage, oedema, inflammatory infiltration, necrosis and fibrosis [22, 28, 40]. Lesions were scored blindly in interstitial, glomerular or tubular structures and using an individual scale of 0 to 28 for extend of histology [40]. Each section was assigned a score between 0 and 4 as follow: 0 (no lesion), 1 (weak), 2 (moderate and localized), 3 (severe and localized), and 4 (severe and diffuse). Criteria scores were assigned for the following criteria: presence of hemorrhage (i), edema (ii), necrosis (iii) or fibrosis (iv), infiltration of lymphocytes (v), macrophages (vi) or polynuclear cells (vii). Scores were then totalized to calculate the structural scores for each individual. Average score for all renal lesions was also calculated for each individual. Leptospires were visualized after silver impregnation following the Warthin-Starry (WS) protocol modified with pyrocatechol [41].

### Immunohistological staining

Immunohistochemical staining of renal leptospires was carried out using an automated stainer (Dako Autostainer Plus) and commercial reagents and kits from Dako system (EnVision FLEX, High pH) according to manufacturer’s instructions. Staining was performed with anti-leptospiral Loa22 monoclonal antibody produced in rabbit (clone E21-4; 1/1000; Antibody Engineering Platform, Institut Pasteur, Paris). Following deparaffinization and rehydration, tissues sections were pre-treated with commercial reagents at pH 9 and incubated with anti-leptospiral antibodies during 20 min. Sections were then incubated with secondary HRP-coupled anti-rabbit antibodies during 20 min. Peroxidase activity was revealed by diaminobenzidine. Slides were counterstained with Harris hemaroxylin solution.

### Purification of DNA

Total DNA was extracted using QIAamp DNA Mini kit (Qiagen). Tissue samples in MagNA Lyser Green Beads tubes were disrupted and homogenized using the MagNA Lyser Instrument (Roche Applied Science) during 50 s at 7,000 r/min. Lysates were incubated at 56°C for 1 h with 190 μL of PBS and 50 μL of proteinase K. After washing steps, the eluted DNA was quantified by spectrophotometry (NanoDrop 2000, ThermoFisher).

### Total RNA extraction and reverse transcription

Total RNA isolation was performed with kits and instruments from Roche Applied Science [42]. Briefly, tissue was placed into MagNA Lyser Green Beads tubes with 400 μL cold lysis buffer and submitted to 2 pulses of 50 s at 800 X g in the MagNA Lyser instrument. Total RNA was isolated from lysates using the spin column-based High Pure RNA Tissue kit. After purification, 50 μL of RNA were treated for 30 min at 37°C with DNase (Turbo DNA-free kit; Ambion, Applied Biosystems). Total RNA (1 μg) was reverse-transcribed into cDNA using random hexamers from Transcriptor First Strand cDNA synthesis kit (Roche Applied Science). The activation step at 25°C for 10 min was followed by the reverse transcription at 55°C for 30 min and enzyme inactivation at 85°C for 5 min.

### Quantitative PCR

Primers and probes (Table 1) were purchased from Eurogentec. Renal DNA was amplified using the LightCycler FastStart DNA Master SYBR Green I and targeted the *lfb1* gene [43] on a LightCycler 2.0 (Roche Applied Science). Alternatively, bacterial load was assessed using the LightCycler 480 Probe Master targeting *lipL32* gene on a LightCycler 480 II instrument (software v.1.5.0; Roche Applied Science) [44]. Genomic DNA from corresponding leptospires was used as a positive control. Quantitative PCR for transcripts of the cytokines IL-1β, IL-10, TNF-α, TGF-β, iNOS, the chemokines IP-10/CXCL10 and MIP-1α/CCL3, and for reference genes glyceraldehyde-3-phospho-deshydrogenase (GAPDH) and β–actin were conducted from cDNA on a LightCycler 480 II using the LightCycler 480 SYBR Green I Master. Each qPCR was carried out with 2 μL of cDNA or gDNA in 20 μL final volume following gene-specific amplification programs (detailed in Table 1) and the specificity of SYBR Green I-based qPCR assays was verified by the melting temperature (T*m*) of the amplicon as calculated by the instrument software (see Table 1). Results were validated only when threshold cycle (Ct) values were under the limit value of 40 cycles and with an acceptable reproducibility between qPCR replicates (less than 5% of variation).

**Table 1:** Details and sequence of primers used for qPCR assays.

Gene	GenBank ^a^	Sequence (5’-3’) ^b^	Size ^c^	Tm ^d^	Efficiency ^e^
Hamster β-actin	AF046210	(F) TCTACAACGAGCTGCG	357	88.33	1.802±0.04
		(R) CAATTTCCCTCTCGGC			
Murine β-actin	NM_007393	(F) AAGAGAAGCTGTGCTATGTT	251	86.25	1.660±0.09
		(R) GTTGGCATAGAGGTCTTTACG			
Hamster MIP-1α/CCL3	AY819019.1	(F) CTCCTGCTGCTTCTTCTA	210	84.97	1.856±0.06
		(R) TGGGTTCCTCACTGACTC			
Murine MIP-1α/CCL3	NM_011337.2	(F) TCAGACACCAGAAGGATAC	159	84.64	1.780±0.03
		(R) CTGAGAAGACTTGGTTGC			
Hamster IP-10/CXCL10	AY007988.1	(F) CTCTACTAAGAGCTGGTCC	150	83.38	1.900±0.06
		(R) CTAACACACTTTAAGGTGGG			
Murine IP-10/CXCL10	NM_021274.1	(F) CTTAACCACCATCTTCCCAA	152	76.62	1.581±0.03
		(R) GATGACACAAGTTCTTCCA			
Hamster GAPDH	DQ403055	(F) CCGAGTATGTTGTGGAGTCTA	170	85.67	1.938±0.04
		(R) GCTGACAATCTTGAGGGA			
Murine GAPDH	NM_008084	(F) TCATCCCAGAGCTGAACG	213	86.39	1.853±0.02
		(R) GGGAGTTGCTGTTGAAGTC			
Hamster IL-1β	AB028497.1	(F) ATCTTCTGTGACTCCTGG	156	85.29	1.852±0.04
		(R) GGTTTATGTTCTGTCCGT			
Murine IL-1β	NM_008361	(F) GTGTGGATCCCAAGCAATAC	161	83.35	1.660±0.09
		(R) GTTGTTTCCCAGGAAGACAG			
Hamster IL-10	AF046210	(F) TGGACAACATACTACTCACTG	308	85.50	1.871±0.05
		(R) GATGTCAAATTCATTCATGGC			
Murine IL-10	NM_010548	(F) ATTCCCTGGGTGAGAAG	259	83.45	1.891±0.04
		(R) CTCTGTCTAGGTCCTGG			
Hamster TNF-α	AF046215	(F) AACGGCATGTCTCTCAA	278	88.05	1.849±0.03
		(R) AGTCGGTCACCTTTCT			
Murine TNF-α	NM_013693	(F) CAACGGCATGGATCTCA	325	87.80	1.832±0.04
		(R) GGACTCCGCAAAGTCT			
Hamster iNOS	NM_001281644	(F) CCATTCTACTACTATCAGGTCG	274	88.4	1.844±0.04
		(R) TGCCCTTGTACTGGTTCAT			
Murine iNOS	NM_010927.4	(F) CCTCATGCCATTGAGTTC	349	88.5	1.920±0.04
		(R) AGTCATGTTTGCCGTC			
Hamster TGF-β1	AF046214	(F) ACGGAGAAGAACTGCT	245	89.7	1.859 ±0.04
		(R) ACGTAGTACACGATGGG			
Murine TGF-β1	NM_011577.2	(F) ACCGCAACAACGCCATCTAT	200	86.01	1.788±0.03
		(R) GTA ACG CCA GGA ATT GTT GC			
*Leptopira lfb1* ^f^	LA0322	(F) CATTCATGTTTCGAATCATTTCAAA ^f^	331	83.40	1.735±0.03
		(R) GGCCCAAGTTCCTTCTAAAAG ^f^			
*Leptospira lipL32* ^g^	-	(F) AAG CAT TAC CGC TTG TGG TG	242	-	1.788±0.03
		(R) GAACTCCCATTTCAGCGATT			
		(P) AAAGCCAG GACAAGCGCCG			

^a^ Accession Number of mRNA sequence in GenBank (NCBI) used for primer design.

^b^ (F), (R) and (P) indicate forward and reverse primer and probe sequences, respectively.

^c^ PCR product size in base pairs.

^d^ PCR product melting temperature (Tm) in °C.

^e^ Efficiency for PCR was determined by elaboration of standard curves as described in Materials and Methods.

^f, g^ As described by Mérien et al. [43] and Stoddard et al. [44], respectively.

### Quantification of leptospires

A standard curve obtained from serial 10-fold dilutions of known numbers of leptospires was used for absolute quantification. Results were expressed as the number of *Leptospira* equivalent genomes per μg of kidney tissue DNA.

### Quantification of cytokine gene expression

The normalization of target gene expression was processed using qBase PLUS software (Biogazelle, Belgium) by extracting the expression levels of the reference genes. The relative normalized expression ratio of target gene was then calculated as the ratio of the expression level in infected individuals to the expression level in control non infected animals used as calibrators.

### Statistical analysis

Statistical studies were performed using GraphPad Prismv4 (GraphPad Software Inc.). Unpaired *t*-test was used for analysis of significant differences in renal *Leptospira* concentrations, grading of histological lesions and cytokine gene expression between animals.

## Results

### Clinical signs, survival and body weight variation in animals infected with virulent B3-13S leptospires

**Fig 1:**
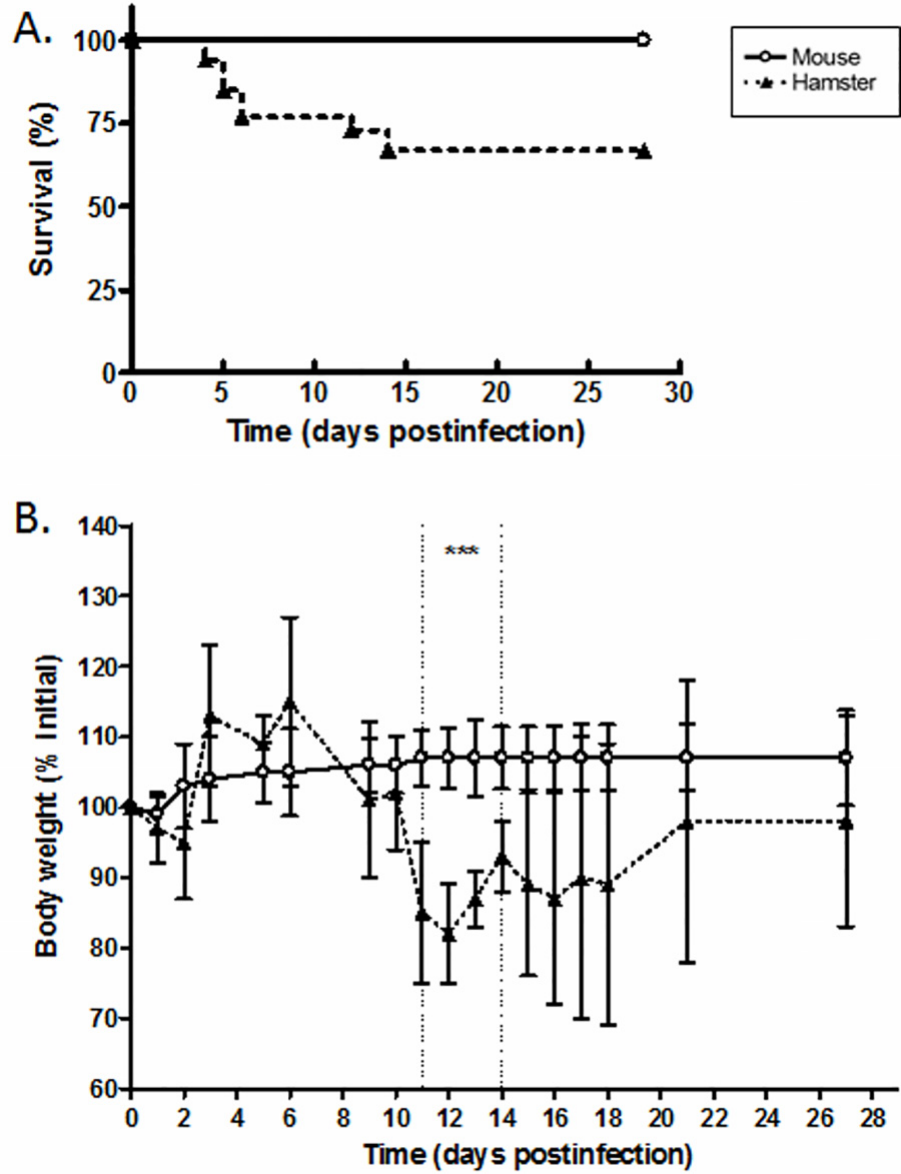
Survival and body weight parameters in hamsters and mice infected with virulent *L*. *borgpetersenii* serogroup Ballum isolate B3-13S. Six- to eight-week-old hamsters or mice were injected with B3-13S isolate at 1 x 10^8^ leptospires and survival (A) and body weight (B) were monitored until 28 days postinfection as described in Materials and Methods. (A) Data represent mean of three independent experiments (*N* = 6, 9, 17). (B) Values represent mean ± SD for hamsters (*N* = 15) and mice (*N* = 10). Significant difference between animals was evaluated using an unpaired *t*-test. ***P<0.0005.

B3-13S-infected mice did not show any signs and symptoms of illness while some hamsters presented prostration, loss of appetite and decreased reactivity during the onset of infection. Survival and variation in body weight in infected animals were monitored to measure clinical signs of the disease until 28 days after inoculation (Fig 1). Survival of mice was confirmed while hamsters presented lethality until 14 dpi with 67% of survival and the highest number of deaths recorded during the acute state of leptospirosis between D4 and D6 postinfection (Fig 1A). Body weight in murine model was not affected by infection compared to initial weight contrasting with variations in hamsters reaching a maximum weight loss of 18% at 12 days postinfection (82 ± 7% of initial weight; Fig 1B). Interestingly, weight variation in hamsters was significantly different compared to mice between D11 and D14 after inoculation (*P* value < 0.0005; Fig 1B). Body weight in hamsters was then restored to initial weight with no difference in variation after 15 days postinfection compared to mice until D28 postinfection.

### Histopathological changes in kidneys during chronic leptospirosis

**Fig 2:**
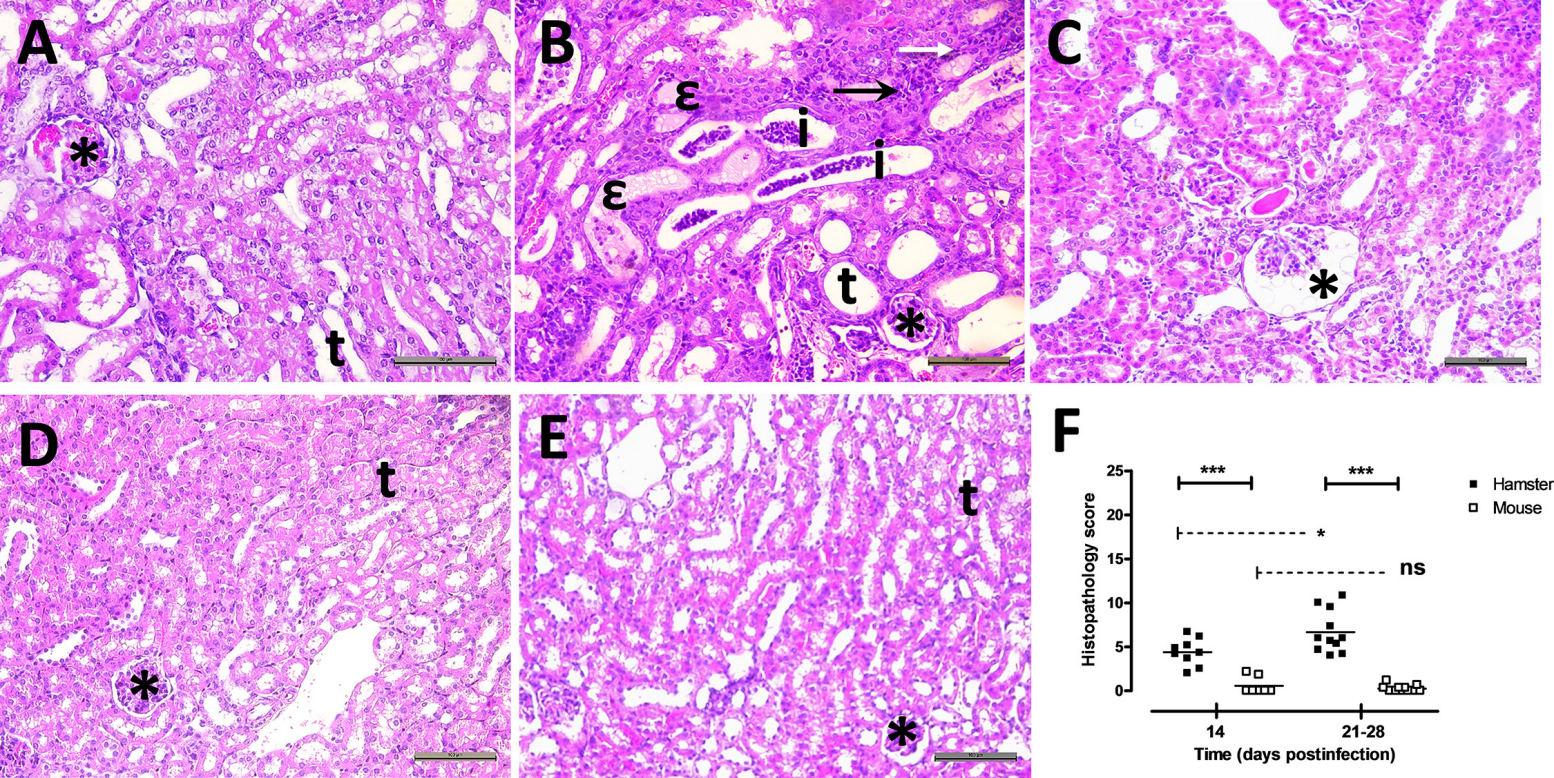
Inflammatory lesions were observed in the kidneys of animals during chronic carriage of virulent *L*. *borgpetersenii* serogroup Ballum isolate B3-13S. (A) Normal glomerulus (*) with typical renal tubules (t) were observed in sections of kidneys collected from non-infected control hamster (HE stain, Magnification, X200). (B) Focal interstitial infiltration of polymorphonuclear cells (filled arrow) and lymphocytes (open arrow) were observed in kidneys at D28 postinfection. Dilatation of tubules (t) was commonly observed with inflammatory infiltration (i) or hyaline deposit (ε) in the lumen, and congestion (*) of several glomeruli. (C) Dilatation of the Bowman’s space (*) was also noticed at D28 postinfection in hamster kidneys. (D) Typical renal structures with glomerulus (*) and tubules (t) in kidneys from control non-infected mouse. (E) Histological observations of renal tissues from mouse at D28 postinfection showing normal glomerulus (*) and tubules (t). (A-E) HE stain. Scale bar represents 100 μm. (F) Lesion score was calculated for each individual at 14 and between 21–28 days postinfection. Values are means (horizontal line) and individual score (dots). Significant difference between animals or time postinfection was evaluated using an unpaired *t*-test. *P<0.05, ***P<0.0005, *ns*: not significant.

Hamsters and mice were infected with 1 x 10^8^ bacteria of the B3-13S isolate. Kidneys were collected from animals at D14, D21 and D28 postinfection and organ sections were stained with HE (Fig 2). Histological observations in hamster kidneys (Fig 2A–2C) confirmed inflammatory foci made of polymorphonuclear neutrophils and lymphocytes surrounding the tubules at D28 postinfection, supporting acute or subacute tubulointerstitial nephritis (Fig 2B). Necrosis of tubular epithelial cells was also observed. At D28 postinfection, all hamsters had a similar pattern of tubular and glomerular damages with massive inflammatory infiltrates in the interstitium (Fig 2B). Luminal dilatations of proximal tubules were seen with massive hyaline deposit (Fig 2B). Glomerular congestion with dilatation of Bowman’s space and disorganisation of mesangial cell structure was also observed (Fig 2C). Excepting weak to moderate interstitial infiltration of lymphocytes, no lesions were observed in mouse kidneys 28 days postinfection (Fig 2D and 2E).

To evaluate morphological alterations in animal kidneys, lesions were graded in the different renal structures (Fig 2F, S1 Fig) to follow the injury course over time and to compare the renal histopathology between hamster and mouse. While no difference was observed in interstitium or glomerular structure of hamster kidneys between 14 or 21–28 days postinfection (S1A and S1B Fig), histopathology score was significantly higher in tubules at the end of experiment (score = 9.8 at D21-28 compared to score = 5.6 at D14; S1C Fig) explaining the increase of totalized lesion score in hamster kidneys (4.4 and 6.7 at D14 and D21-28, respectively; Fig 2F). In contrast, no evolution of lesions in mouse kidneys was showed during experimental infection. The comparison of the total histopathology score between animal models is significantly higher in hamster compared to mouse kidneys at D14 and D21-28 postinfection (*P* value < 0.0005; Fig 2F) reflecting more renal damages in hamsters while weak lesions were observed in mouse kidneys during chronic leptospirosis.

### Development of fibrosis in kidneys of infected animals and regulation of renal TGF-β expression during chronic leptospirosis

**Fig 3:**
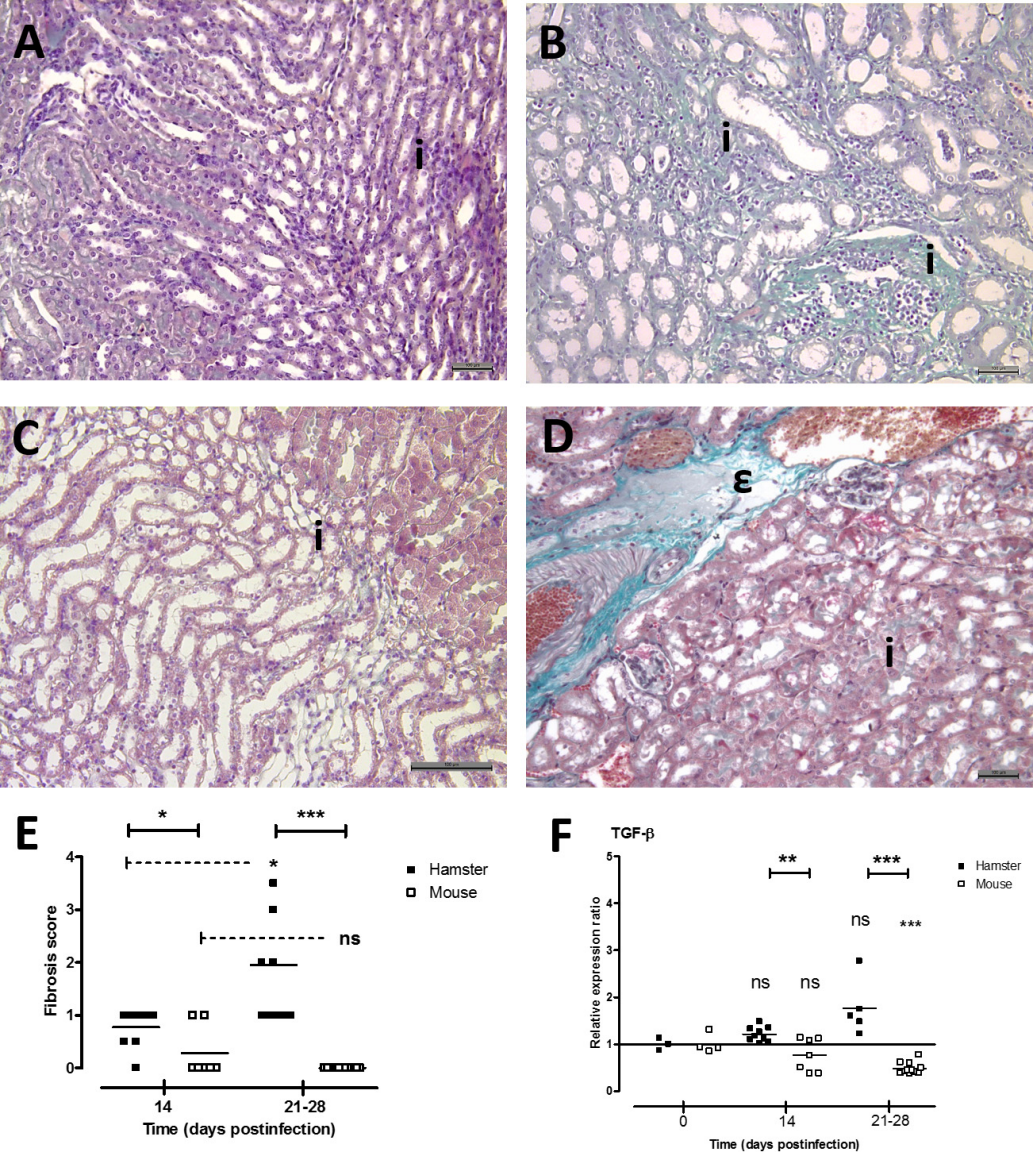
Development of fibrosis in hamster kidneys during chronic leptospirosis. Observation of kidneys from control non-infected hamster (A) and mouse (C) showing normal staining for interstitial structures. (B) Intense staining of the collagen was observed in the interstitium (i) of kidneys from hamster inoculated with B3-13S isolate at D28 postinfection. (C) Intense normal staining of blood vessels (ε) contrasting with no staining of interstitium (i) in mouse kidneys 28 days after infection with B3-13S leptospires. (A-D) Masson’s trichrome stain, bar represents 100 μm. (E) Score of renal fibrosis at 14 and between 21 and 28 days postinfection in hamsters and mice inoculated with virulent B3-13S isolate. (F) Gene expression of cytokine TGF-β in kidneys from infected animals was quantified by RT-qPCR as described in Materials and Methods. (E-F) Data are means (horizontal line) and individual value (dots). Significant difference between animals or time postinfection was evaluated using an unpaired *t*-test. *P<0.05, *P<0.005, ***P<0.0005, *ns*: not significant.

Concomitant to evaluation of inflammatory lesions in kidneys, development of renal fibrosis was investigated in animals during chronic leptospirosis (Fig 3). Masson’s trichrome staining showed evidences of interstitial fibrosis in hamster kidneys (Fig 3B) contrasting with no fibrotic lesion in mouse kidneys except 2 / 7 mice with low score fibrosis at 14 days postinfection (individual score = 1 and mean score = 0.28; Fig 3E). Scoring of fibrosis in animals revealed mild fibrosis in hamsters at D14 postinfection that significantly increased between 21 and 28 days postinfection (score = 1.95; Fig 3E). As known to be related to fibrosis, gene expression of TGF-β was quantified in kidneys of infected animals (Fig 3F). Interestingly, TGF-β transcript level was poorly regulated in hamsters with no significant difference at D14 and D21-28 postinfection compared to initial level. In contrast, TGF-β gene expression was significantly downregulated in mouse kidneys at 28 days postinfection (relative expression ratio = 0.48) compared to control level.

### No difference in bacterial load between infected animals

**Fig 4:**
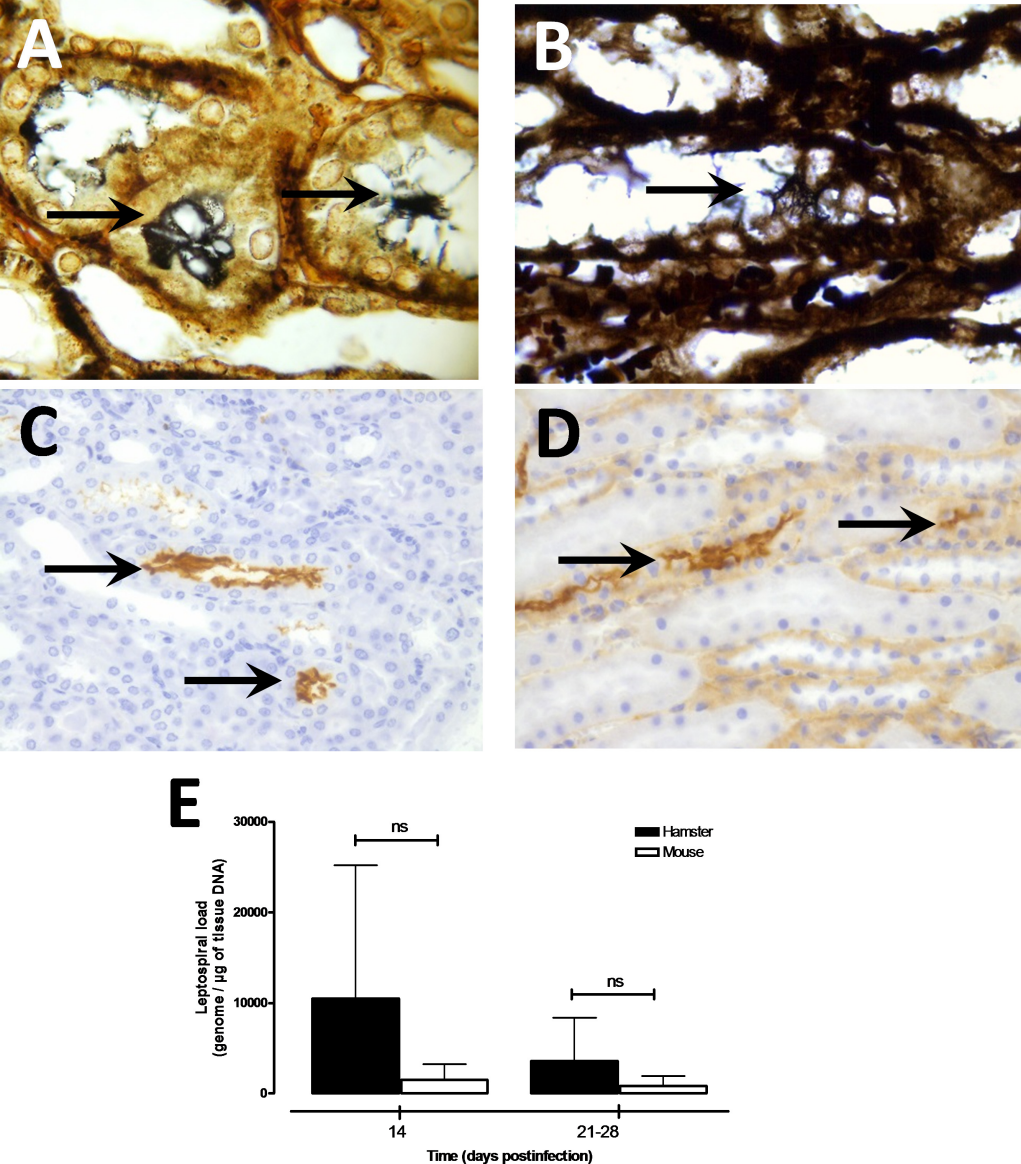
Detection of leptospires and quantification of bacterial load in the kidneys of experimentally infected animals during carrier state. Modified Warthin-Starry silver staining was used for spirochetes observation in the kidneys of hamsters (A) and mice (B) euthanized at 28 days postinfection. Note the typical form of spirochetes with terminal hooks found in tubular structures (arrows). Magnification, X1,000. Immunohistochemical staining was performed as described in Materials and Methods to confirmed presence of leptospires in kidneys from infected hamsters (C) and mice (D). Intense brown coloration of leptospires was observed in lumen of tubular structures (arrows). Magnification, X400. (E) Quantification of bacterial load was quantified in kidneys from hamsters (filled squares) and mice (open squares) infected intraperitoneally with 1 x 10^8^ virulent isolate B3-13S (*L*. *borgpetersenii* serogroup Ballum). Genomic DNA was extracted from renal tissue collected at 14 and 21–28 days postinfection and leptospires were detected by qPCR assays as described in Materials and Methods. Results are expressed as *Leptospira* genomes per μg of kidney tissue DNA. Values are means (horizontal line) and individual load (dots). Significant difference in the bacterial loads was evaluated using an unpaired *t*-test. *ns*: not significant.

Presence of leptospires was confirmed in kidneys of hamsters and mice chronically infected with virulent B3-13S leptospires using WS and IHC staining (Fig 4A–4D). Leptospires were not seen in the interstitium or between cells but large clusters of bacteria were localized in the tubules of infected animals. Quantitative PCR targeting leptospiral genes confirmed the presence of B3-13S leptospires in mouse and hamster kidneys until 28 days postinfection, and leptospires were quantified in the kidneys of animals at 14, 21 and 28 days after inoculation (Fig 4E). No statistical difference was observed in the bacterial concentration between hamsters and mice infected with B3-13S neither at day 14 (approx. 10,400 and 2200 *Leptospira* equivalent genome / μg of tissue DNA in hamsters and mice, respectively) nor at between day 21 and 28 after infection (4060 and 1178 / μg of tissue DNA in hamsters and mice, respectively). High variability was observed in bacterial load especially in hamster kidneys possibly reflecting disparity in leptospire repartition in tissue. Leptospiremia and bacterial load were also investigated presenting absence of circulating leptospires in blood, lungs and liver at 14 days postinfection (data not shown).

### Differential regulation of the cytokine and chemokine gene expression in kidneys of infected animals during chronic carriage of *Leptospira*

**Fig 5:**
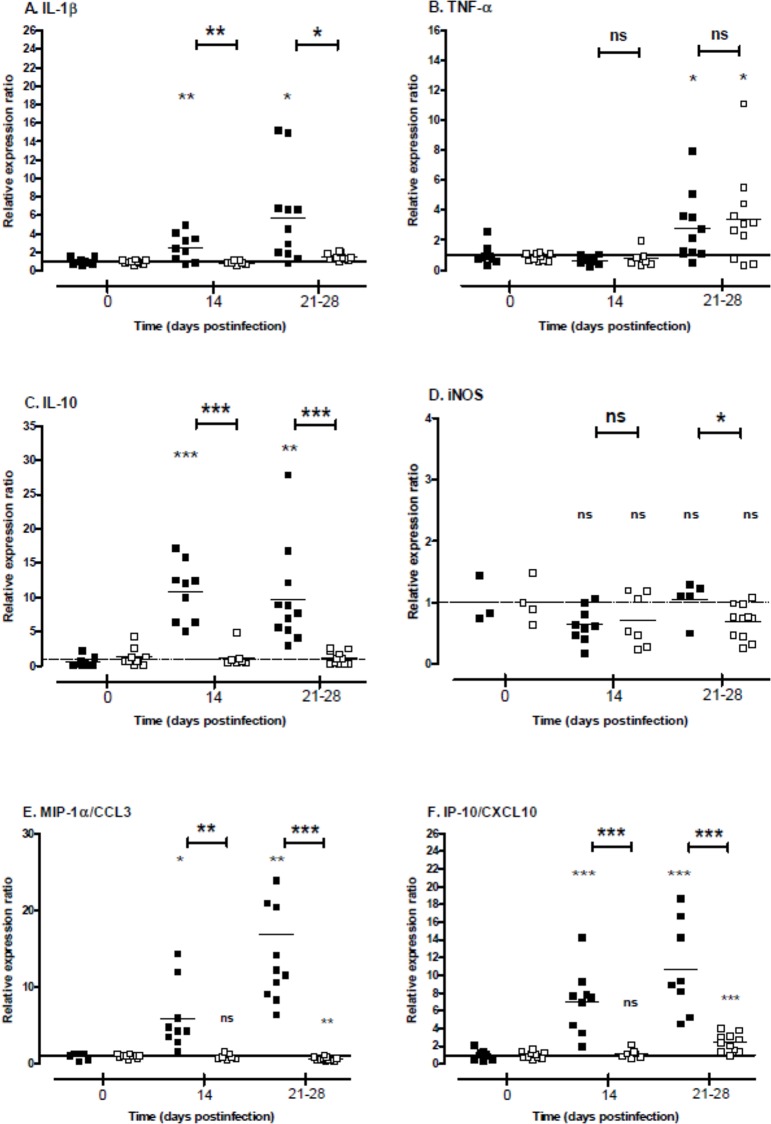
Cytokine, iNOS and chemokine gene expression in the kidneys depending on infected animals during *Leptospira* carrier state. Mice (open squares) and hamsters (filled squares) were infected with a sublethal dose of *L*. *borgpetersenii* serogroup Ballum isolate B3-13S. Total mRNA was extracted from kidneys at 14 and between 21 and 28 days postinfection and RT-qPCR assays were processed as described in Materials and Methods. Results are expressed as expression ratio relative to non-infected animal controls (time 0). Values are means (horizontal line) and individual ratio (dots). Significant difference in the gene expression levels were evaluated between animals and compared to control individual using an unpaired *t*-test. *P<0.05, **P<0.005, ***P<0.0005, *ns*: not significant.

Gene expression of cytokines IL-1β, IL-10 and TNF-α and of iNOS was quantified in the kidneys of animals 14 and between 21 and 28 days postinfection compared to control animals at time of inoculation and using RT-qPCR technique (Fig 5). IL-1β expression was significantly up-regulated in hamster kidneys at D14 (ratio = 2.5) and D21-28 (5.8) compared to control, while it was not regulated in mouse kidneys (ratios < 2; Fig 5A). Expression levels of IL-1β were significantly higher in hamsters compared to mice. TNF-α expression showed significant increase in both animal models at D21-28 (2.7 and 3.4 in hamster and mouse kidneys, respectively; Fig 5B). No difference was noticed in the expression levels of this cytokine between hamsters and mice. Expression profiles of IL-10 presented a high up-regulation in hamster kidneys at D14 and D21-28 (ratios = 10.8 and 9.7, respectively) contrasting with the absence of regulation in mouse kidneys compared to non-infected animals (Fig 5C). These results correlate with a significant difference in the regulation of IL-10 expression between animal models. Surprisingly, expression level of iNOS was not significantly regulated in both animal models neither at 14 nor at 21–28 days postinfection (ratios ≥ 0.7 for both models; Fig 5D).

The expression level of chemokines MIP-1α/CCL3 and IP-10/CXCL10 was also quantified in kidneys of animals experimentally infected (Fig 5E and 5F). Both chemokines were markedly overexpressed in hamster kidneys at D14 and D21-28 postinfection compared to control animals (ratios = 16.82 and 10.7 for MIP-1α/CCL3 and IP-10/CXCL10 in hamsters at D21-28, respectively). In contrast, while IP-10/CXCL-10 was also up-regulated (ratio = 2.4 at D21-28 postinfection), MIP-1α/CCL3 expression was significantly down-regulated in mouse kidneys (0.6 at D21-28 postinfection). Comparison between both animal models showed high significant differences in expression level at D21-28 for MIP-1α/CCL3 and IP-10/CXCL10 (*P* value < 0.0005).

## Discussion

Using a virulent *L*. *borgpetersenii* Ballum isolated from a natural mouse reservoir, we studied the pathophysiology of the renal leptospirosis in infected animal models during the chronic state of the disease and showed differences in the development of renal lesions between the hamsters comparatively to the asymptomatic murine reservoir. First, lethality and clinical signs of the disease with decrease of body weight were observed in infected hamsters until 14 days postinfection correlating with similar observation in a sublethal murine model of leptospirosis infection [45] or with body weight variations in hamsters infected with another *L; borgpetersenii* serovar [46]. Hamsters then apparently regained a clinically healthy state with initial body weight but histological observations of kidneys revealed pathological changes. Indeed, tubulointerstitial nephritis, glomerular congestion and fibrosis were observed in infected hamsters, while no or slight lesions (essentially weak interstitial infiltration of lymphocytes) were seen in mice. Interstitial nephritis is a common morphological alteration observed in classical reservoirs as rats [28] and mice [47, 48]. Likewise, commonly susceptible guinea pigs that became chronic carrier of virulent leptospires showed interstitial nephritis, but also presented glomerular alterations with atrophy of the normal structures and hyaline deposit in the tubular lumen [20]. Interstitial nephritis and infiltration of mononuclear cells around the glomeruli were also reported in chronically infected cattle related to white-spotted pattern of kidneys [49, 50]. This is in accordance with the interstitial lesions observed in the kidneys of hamsters chronically infected with B3-13S, associated with necrosis of tubular epithelial cells and glomerular modifications (Fig 2). Similarly, interstitial inflammation and focal necrosis of tubular epithelium were observed in kidneys during acute human leptospirosis with tubular hyaline staining [51]. Glomeruli can also be affected with hyperplasia of mesengial cells and infiltration of inflammatory cells [52], and glomerulonephritis was also noticed in human cases [53]. Renal fibrosis was previously reported in cases of leptospirosis-related human CKD [9, 10]. In our experiments, renal fibrosis was also observed in hamsters, but not in OF1 mice, contrasting with previous observations of mild renal fibrosis in C57BL/6J mice infected with *L*. *interrogans* serovar Copehageni strain Fiocruz L1-130 [35, 48]. This discrepancy might possibly be due to difference in *Leptospira* and mice strains used for experimental infection. Indeed, B3-13S leptospires were isolated from kidneys of captured reservoir mouse and identified as a Ballum serovar [37]. Moreover, OF1 outbred mice previously showed high resistance to leptospiral infection [54] underlying specificity in evolution of disease outcome and pathological alterations depending on precise host-pathogen pair to be considered during chronic leptospirosis. Expression of pro-fibrotic TGF-β was quantified and showed significant decrease in mouse kidneys while not regulated in hamster kidneys (Fig 3). Previous studies on *Leptospira*-induced fibrosis in mouse models also reported absence of TGF-β regulation in kidneys during chronic carriage of bacteria [35, 48].

Regarding the differences in the lesions, we quantified the bacterial load of leptospires in the kidneys during chronic carriage but no difference was observed. Localization of the bacteria was observed using WS and IHC staining of leptospires with large clusters of spirochetes confined into the tubular lumen in kidneys of infected animals as previously shown in rodent reservoirs [35, 55]. Aggregation of pathogenic leptospires may be assimilated to biofilms as previously observed *in vitro* [56] and was proposed as a survival mechanism in the natural environment [57]. A recent study showed the production of an active collagenase by *L*. *interrogans* strain Lai in the presence of human renal tubular epithelial cells, that can hydrolyse different types of human collagens [58] possibly leading to the activation of tubular cells in the kidneys and possible induction of inflammatory markers during the chronic carriage of the bacteria.

Cytokine gene expression was investigated in the kidneys during the carrier state of animals, and results showed that pro-inflammatory IL-1β and TNF-α were up-regulated in both experimental models. However, expression levels of IL-1β and IL-10 but not TNF-α were significantly different between mice and hamsters. Interleukin-10 is known to regulate inflammatory processes by inhibiting the expression of pro-inflammatory cytokines as TNF-α and IL-1β and, thus, this cytokine also protects from deleterious tissue lesions related to massive cytokine expression [59, 60]. Overexpression of IL-10 was previously noticed in kidneys of mice challenging development of renal fibrosis during chronic leptospirosis [48]. Deficiency in IL-10 expression leads to exacerbate glomerulonephritis in transgenic mice [61]. Consequently, up-regulation of IL-10 expression may compensate the overexpression of inflammatory cytokines playing a role against local inflammatory processes and avoiding acute renal lesions in hamster kidneys. Inflammatory TNF-α was previously investigated in kidneys of three mouse models challenging renal alterations after leptospirosis infection [62]. Transcript level was shown to be upregulated following the early days postinfection and maintained until one week after inoculation. However, we also quantified overexpression of inflammatory TNF-α in kidney from chronically infected animals but no difference was observed between models (Fig 5). Interestingly, though not lethal, mice deficient for production of TNF-α receptor TNFR presented more renal lesions compared to WT mice when challenging *Leptospira* infection [63] suggesting a protective effect of this cytokine during chronic leptospirosis. Role of iNOS in resolution of leptospirosis was previously investigated and suggested its involvement in survival against leptospirosis [34] but also in appearance of renal fibrosis in infected mice [35]. However, no regulation of iNOS gene expression in kidneys was observed in hamsters that developed renal fibrosis during carriage of leptospires (Fig 3).

Regulation of chemokines MIP-1α/CCL3 and IP-10/CXCL-10 was also investigated and significant differences in the transcript level were reported in the kidneys of animals. MIP-1α/CCL3 was overexpressed in hamster while significantly down-regulated in mouse kidneys. Previous study also showed an overexpression of MIP-1α/CCL3 in the kidneys of hamster 4 days after infection with a virulent *Leptospira* strain [64]. MIP1-α/CCL3 belongs to the CC chemokine family that act on recruitment of several leukocytes [65], and up-regulation of this chemokine was suggested to be responsible for the recruitment of leukocytes in glomeruli in patients with glomerulonephritis [66]. Blockade of MIP-1α/CCL3 receptor CC chemokine receptor CCR1 by antagonist molecule reduced interstitial infiltration of macrophages and T lymphocytes and renal fibrosis in kidneys of mice developing adriamycin-induced nephropathy with tubulointerstitial injury [67]. Thus, overexpression of this chemokine could explain the infiltration of immune cells observed in tissue lesions in hamster kidneys contrasting with possible effect of its down-regulation in mice related with slight inflammatory lesions. The chemokine IP-10/CXCL10 is a member of the CXC chemokine family [68] and is known to be produced by glomerular cells, tubular epithelial cells and interstitial fibroblasts [69, 70]. Moreover, its up-regulation is related to interstitial fibrosis and nephritis in rats [70] and to human glomerulonephritis [71]. IP-10/CXCL-10 transcript level was previously quantified in kidneys from hamsters infected with virulent leptospires and showed an increase of the gene expression during the acute phase of infection [64, 72]. Herein, IP-10/CXCL10 was also overexpressed in the kidneys of both animal models during the carrier state, with a higher up-regulation in hamsters compared to mice. Considering the chemotaxis activity of IP-10/CXCL10 via its receptor CXCR3 on leucocytes including macrophages recruitment into kidneys [73], IP-10/CXCL10 overexpression may possibly be related to interstitial infiltration of leucocytes in renal tissues from animals chronically infected with B3-13S. Interestingly, IP-10 neutralization by monoclonal antibody or CXCR3 deficiency in a transgenic mouse model were associated with increased severity of renal fibrosis in a unilateral ureteral obstruction model related with overexpression of TGF-β in kidneys [74]. Thus, contrasting with significant downregulation of chemokine MIP-1α/CCL3, increase in IP-10/CXCL10 transcript level in mice might possibly be related to protective antifibrotic effect in mouse model through negative regulation of TGF-β expression. However, similar pattern would not be applicable in hamster model.

Both MIP-1α/CCL3 and IP-10/CXCL10 chemokines are important chemotaxis potents on leucocytes and murine models of the disease showed infiltration of CD11b+ macrophages and CD3+ lymphocytes in renal tissues during chronic leptospirosis [35, 45]. However, use of CD3 ko mice presented evidences that T- and B-cells are not involved in progression of fibrotic lesions in kidneys [35].

Recently, concept of M1/M2 macrophage polarization emerged with macrophages’ unique ability to make polar-opposite repair/heal (M2) or kill/inhibit (M1) type responses [75]. Furuichi and coll. described interesting findings regarding chemokine cascades involved in progression of CKD from AKI suggesting that IP-10/CXCL10-producing macrophages mediate regeneration and resolution of tubular necrosis while interstitial fibrosis was related to action of CXC3R1 (fractalkine receptor) on macrophages [76]. Phenotype of resident and infiltrating macrophages in kidneys was suggested at playing a major role in progression or resolution of renal inflammation and fibrosis [77]. Indeed, M1 phenotype was associated with inflammatory processes and renal necrosis that exacerbate renal cell damage while M2 phenotype was related to tissue remodeling and profibrotic effects in kidneys. Interestingly, macrophage subtypes were classified depending on their surface markers and cytokines produced and showed M1 phenotype characterized by TNF-α and IL-1β cytokine production while M2 macrophages were related to IL-10, TGF-β and TNF-α excretion [78]. Transition from inflammatory M1 phenotype to wound healing/profibrotic M2 phenotype induces the progression from the inflammation phase to the tissue repair phase and also promote remodeling stage [78]. However, dysregulation of M2-type cells could potentially display increased production of type I and type III collagens and participate in fibrotic process. Putting together our findings on differential gene expression related to distinct lesion profiles in kidneys of animals during chronic leptospirosis, possible involvement of particular subset of macrophage might explain resolution or progression of renal injuries and CKD during leptospirosis. Imbalance in M1 and M2 phenotype concentration in renal microenvironment may possibly occur in susceptible host contributing to deleterious inflammatory and fibrotic renal lesions.

To summarize, the present study highlights differential development of histological lesions in kidneys during the carriage of pathogenic leptospires in animals depending on their common reservoir (slightly or not affected) or incidental carrier (marked morphological changes) status. Results suggest adverse evolution in hamster renal pathophysiology associated with an increase of the gene expression of inflammatory mediators. Thus, variability in the regulation of the cytokine and chemokine gene expression could explain the discrepancy observed in the renal tissues between animal models, possibly favoring local infiltration of inflammatory or fibrotic macrophages in hamster kidneys leading to lesions corresponding to those observed during leptospirosis-CKD in human cases.

## Supporting Information

S1 Fig Grading of histological lesions showed differential scores in kidneys of animals chronically infected with the virulent *L*. *borgpetersenii* serogroup Ballum isolate B3-13S. Sections of kidneys collected from hamsters (filled squares) and mice (open squares) during the carrier state at D14 or between D21 and D28 postinfection were HE stained for histological observation. (A—C) Interstitial, glomerular and tubular structures were scored as detailed in Materials and Methods for haemorrhage, oedema, inflammatory infiltration, necrosis and fibrosis. Significant difference between animals or time postinfection was evaluated using an unpaired *t*-test. *P<0.05, **P<0.005, ***P<0.0005, *ns*: not significant.
(TIF)
