# Supplementary figures and images for: Cytokine and Chemokine Expression in Kidneys during Chronic Leptospirosis in Reservoir and Susceptible Animal Models
Source: PLoS One. 2016 May 24;11(5):e0156084. doi: 10.1371/journal.pone.0156084 (PMC4878748; doi:10.1371/journal.pone.0156084)

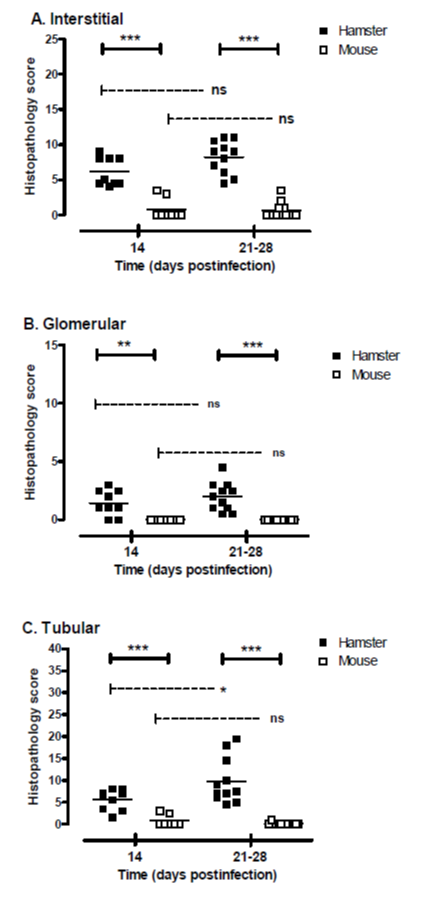

Supplement: S1 Fig — Sections of kidneys collected from hamsters (filled squares) and mice (open squares) during the carrier state at D14 or between D21 and D28 postinfection were HE stained for histological observation. (A—C) Interstitial, glomerular and tubular structures were scored as detailed in Materials and Methods for haemorrhage, oedema, inflammatory infiltration, necrosis and fibrosis. Significant difference between animals or time postinfection was evaluated using an unpaired t-test. *P<0.05, **P<0.005, ***P<0.0005, ns: not significant. (TIF) [file pone.0156084.s001.tif]
